# Cohort study of Western Australia computed tomography utilisation patterns and their policy implications

**DOI:** 10.1186/s12913-014-0526-0

**Published:** 2014-11-05

**Authors:** David AJ Gibson, Rachael E Moorin, C D’Arcy J Holman

**Affiliations:** School of Population Health, University of Western Australia, Perth, Australia; Faculty of Health Sciences, Curtin University, Perth, Australia; Centre for Health Services Research, School of Population Health (M431), The University of Western Australia, 35 Stirling Hwy, Crawley, WA 6009 Australia

**Keywords:** Computed tomography, CT, Health service utilisation

## Abstract

**Background:**

Computed tomography (CT) scanning is a relatively high radiation dose diagnostic imaging modality with increasing concerns about radiation exposure burden at the population level in scientific literature. This study examined the epidemiology of adult CT utilisation in Western Australia (WA) in both the public hospital and private practice settings, and the policy implications.

**Methods:**

Retrospective cohort design using aggregate adult CT data from WA public hospitals and Medical Benefits Schedule (MBS) (mid-2006 to mid-2012). CT scanning trends by sex, age, provider setting and anatomical areas were explored using crude CT scanning rates, age-standardised CT scanning rates and Poisson regression modelling.

**Results:**

From mid-2006 to mid-2012 the WA adult CT scanning rate was 129 scans per 1,000 person-years (PY). Females were consistently scanned at a higher rate than males. Patients over 65 years presented the highest scanning rates (over 300 scans per 1,000 PY). Private practice accounted for 73% of adult CT scans, comprising the majority in every anatomical area. In the private setting females predominately held higher age-standardised CT scanning rates than males. This trend reversed in the public hospital setting. Patients over 85 years in the public hospital setting were the most likely age group CT scanned in nine of ten anatomical areas. Patients in the private practice setting aged 85+ years were relatively less prominent across every anatomical area, and the least likely age group scanned in facial bones and multiple areas CT scans.

**Conclusion:**

In comparison to the public hospital setting, the MBS subsidised private sector tended to service females and relatively younger patients with a more diverse range of anatomical areas, constituting the majority of CT scans performed in WA. Patient risk and subsequent burden is greater for females, lower ages and some anatomical areas. In the context of a national health system, Australia has various avenues to monitor radiation exposure levels, improve physician training and modify funding mechanisms to ensure individual and population medical radiation exposure is as low as reasonably achievable.

## Background

Computed tomography (CT) scanning is considered to be a relatively high radiation dose diagnostic imaging modality [1]. In response to the growing utilisation of CT imaging [2,3], many nations have established or are in the process of implementing guidance and reference for the radiation dose delivered to the patient by CT examinations [4–7]. The risk of cancer from CT scans and how best to incorporate these risks into clinical decisions are under review around the world [8–13]. However, discussion around funding mechanisms, access parameters, the consequent incentives and disincentives the organisation of health systems generate for undertaking these examinations has been lacking.

The increased availability and technological advancements of CT machines have resulted in growth and diversification of CT use well beyond the early days of in-patient head scans and cancer diagnosis [2,14–16]. A report from the United Nations Scientific Committee on the Effects of Atomic Radiation (UNSCEAR) showed CT represented only a few per cent of diagnostic procedures in developed nations, but was responsible for almost one half of diagnostic medical radiation exposure, and is the largest man-made source of ionising radiation exposure in the world [17]. The Organisation for Economic Co-operation and Development (OECD) has reported substantial increases in the per capita rate of CT scanning across the majority of developed countries [2]. The OECD noted increases in the per capita CT scanning rate of 34% in France and 32% in the USA between 2006 and 2010, while hospital CT scanning rates increased by 46% in the United Kingdom (UK) and 10% in Canada between 2006 and 2010, and by 11% in Ireland between 2009 and 2011 [2]. Interpretation of Australian CT scanning trends is complicated by the nation’s mixed health funding arrangements; however, the OECD found an 18% increase in non-public hospital CT scans from 2007 to 2011 [2] and reported Australia’s non-public hospital CT scanning rate was 93.9 scans per 1,000 person-years (PY) compared with the OECD average of 131.8 scans per 1,000 PY in 2011 [18]. Other research has also noted an increasing utilisation of CT in Australia [3].

The bulk of Australia’s non-public hospital CT (out-patient, private clinics and private hospitals) is subsidised by the federal government’s Medicare Benefits Schedule (MBS), which also subsidises general practitioner (GP), pathology, other diagnostic, medical specialist and allied health services for all Australians on a fee-for-service basis. These are the CT services included within the OECD’s estimates and are commonly performed at the request of a GP. CT scans performed within Australia’s public hospitals are included within funding agreements established between the federal and state governments (who operate public hospitals) not under the MBS. The availability of public hospital CT data varies from state to state, leaving an incomplete picture of CT use in Australia.

CT Diagnostic Reference Levels (DRL) are the most common tool used by countries or large health systems to partially homogenise inter-practice radiation dose for the same examination by encouraging providers who exceed DRL parameters to reduce dose levels. However, DRL methodology relies on limited surveys of 10 to 20 ‘standard’ CT scans on ‘typical’ patients by broad anatomical areas (e.g. Australian National DRL) or common clinical indications (UK DRL) voluntarily submitted by providers. Information collected during the DRL surveys does not include CT use epidemiology. Thus most national reports on CT radiation levels lack detail of who is being scanned (males or females, younger or older), the anatomical regions exposed and clinical indications for the scans [1]. With respect to guidelines for use of CT in Australia, there is no binding or regulatory scheme, although the Western Australian Department of Health has developed ‘Diagnostic Imaging Pathways’ guidelines to assist physicians in appropriate use of multiple radiological modalities [19].

When CT is used across the primary and tertiary care sectors as extensively as in Australia, universal DRLs lack context and risk irrelevance to either sector without differentiating their respective applications. Given the difference in the roles of public hospitals and the MBS subsidised services of the private and outpatient sector, it is reasonable to expect the profile of patients receiving CT scans would vary substantially across the two sectors. Subsequently the risk-benefit profiles of patients would also vary substantially when the interactions of machine output, patient sex, age, anatomical area and clinical indication(s) are taken into account [20]. These factors conspire to create unique patient risk profiles to be weighed against similarly unique potential benefits. Epidemiological profiles of system utilisation are essential to understand the differences between provider settings and the demands patients and clinicians are making of CT scans, but are currently lacking within the literature.

This study uses Western Australian (WA) CT utilisation data from the public hospital and MBS-supported settings to examine the epidemiology of adult CT scanning and explore the differences between the two service settings across sex, age and anatomical area to inform future policy options.

## Methods

### Study design and data sources

The study used a retrospective cohort design with three data sources to capture WA CT utilisation and population data from 1 July 2006 to 30 June 2012 (2006/07 to 2011/12 fiscal years):

- MBS billing data on adult CT scans provided aggregate counts of CT scans billed by out-of-hospital and private hospital providers [21]. The aggregate counts were categorised by sex, age group, year of claim and MBS item number. The data for patients aged 15 to 24 years were split by proportion to 18 to 24 years for comparability with public hospital data which was limited to patients 18 years and older.
- WA Department of Health public hospital adult CT scan data (inpatient, outpatient/clinic, emergency, private (Medicare), other/unknown) aggregated by year, gender, age group and scanning procedure code.
- Australian Bureau of Statistics (ABS) Census data provided population counts by gender, age and state each year [22].

### Rate of CT utilisation

Crude rates of adult CT scans per 1,000 PY were calculated according to gender and age group in each year and for the entire study period (number of scans in population/number of people in population × 1000). Population counts provided by the ABS provide the relevant population count at 30th June in each study year.

Age-standardised adult CT scan rates (per 1,000 PY) were calculated, using the direct method in STATA IC, according to gender, anatomical area scanned, and provider setting in each year for the study period.

### Adjusted CT utilisation likelihood

Adjusted incidence rate ratios (IRR) of adult CT scans were estimated using Poisson regression (in STATA IC) following assessment of data for model appropriateness. Adult CT scans of each anatomical area within each provider setting were modelled separately by age group (patients age 85+ years provided the reference group), adjusted for sex and year.

### Ethics

This research was conducted with ethics approval from multiple ethics committees. Approval was given by the Government of Western Australia Department of Health Human Research Ethics Committee (project number #2011/97), the Curtin University Human Research Ethics Committee (project number SMEC-80-10) and The University of Western Australia Human Research Ethics Committee (project number RA/4/1/1785). Informed consent was not required or collected as the data used in this study is collected routinely and aggregated.

## Results

Public hospital CT scans could be performed under several ‘admission’ types: inpatient services were the most common (43%), outpatient/clinic (30%) and emergency patients (26%) were the next most frequent; while only 1% of public hospital services were undertaken on private patients and thus billed directly to Medicare (data not shown). The MBS CT scans, with the exception of the small number performed by a public hospital, were all conducted in private radiological clinics, which may or may not have an affiliation with or proximity to a hospital (public or private).

### Rate of CT scanning in Western Australia

Table 1 presents the number and crude rate of CT scans performed in WA from mid-2006 to mid-2012 by sex and age group in each financial year and over the total study period. The crude adult scan rate increased from 124.8 to 138.0 CT scans per 1,000 PY, with an average of 128.8 CT scans per 1,000 PY for the study period.

**Table 1:** **Western Australia CT scan numbers and crude rates by sex and age group across the study period**

	**2006/07**		**2007/08**		**2008/09**		**2009/10**		**2010/11**		**2011/12**		**Total**	
	**n**	Rate*	**n**	Rate*	**n**	Rate*	**n**	Rate*	**n**	Rate*	**n**	Rate*	**n**	Rate*
**Sex**														
Female	101,517	130.2	106,328	132.5	112,124	135.2	112,083	130.8	113,345	129.0	128,893	142.7	674,290	133.5
Male	93,353	119.4	97,791	121.5	104,716	125.7	105,192	122.0	107,625	121.9	120,764	133.3	629,441	124.1
Total	194,870	124.8	204,119	127.0	216,840	130.5	217,275	126.4	220,970	125.4	249,657	138.0	1,303,731	128.8
**Age group**														
18-24 years	6,772	32.6	7,234	33.6	7,989	35.5	7,718	32.9	7,764	32.7	8,772	36.6	46,249	34.0
25-44 years	39,724	66.7	40,817	66.8	43,018	68.1	42,003	64.1	41,360	61.5	48,438	69.9	255,360	66.2
45-64 years	74,026	143.5	77,160	145.3	81,120	148.0	82,029	145.6	82,687	143.2	92,803	156.9	489,825	147.2
65-84 years	63,343	294.7	67,925	307.1	73,254	322.9	74,466	317.7	77,996	322.0	87,269	348.2	444,253	319.6
85+ years	11,005	400.6	10,983	380.0	11,459	380.0	11,059	351.4	11,163	339.7	12,375	357.7	68,044	366.9
Total	194,870	124.8	204,119	127.0	216,840	130.5	217,275	126.4	220,970	125.4	249,657	138.0	1,303,731	128.8
**Provider setting**														
MBS	145,804	93.4	150,747	93.8	159,136	95.7	159,470	92.8	158,671	90.1	182,730	101.0	956,558	94.5
Public Hospital	49,066	31.4	53,372	33.2	57,704	34.7	57,805	33.6	62,299	35.4	66,927	37.0	347,173	34.3
Total	194,870	124.8	204,119	127.0	216,840	130.5	217,275	126.4	220,970	125.4	249,657	138.0	1,303,731	128.8

*Crude rate given in CT scans per 1,000 person-years.

Females were consistently scanned at a higher rate than males in each year. The 2011/12 female CT scan rate of 142.7 per 1,000 PY was 10% higher than the 2006/07 rate. Males presented a similar trend, increasing from 119.4 CT scans per 1,000 PY in 2006/07 to 133.3 CT scans per 1,000 PY in 2011/12.

A wide range of CT scanning rates was observed across age groups. A steep gradient from younger to the oldest adults was notable throughout the study period. The scanning rate of patients 18–24 years of age increased by 5% and the scanning rate of patients aged 25–44 years increased by 12% from 2006/07 to 2011/12. The greatest variation over the study period occurred in the two oldest age groups, albeit with different patterns. The CT scanning rate in patients aged 65–84 years increased by 18% to a 2011/12 rate of 348.2 scans per 1,000 PY. However, the rate in those aged 85+ years began higher at 400.6 scans per 1,000 PY and then declined by 11% over the study period.

### Division of CT services across provider settings

**Figure 1:**
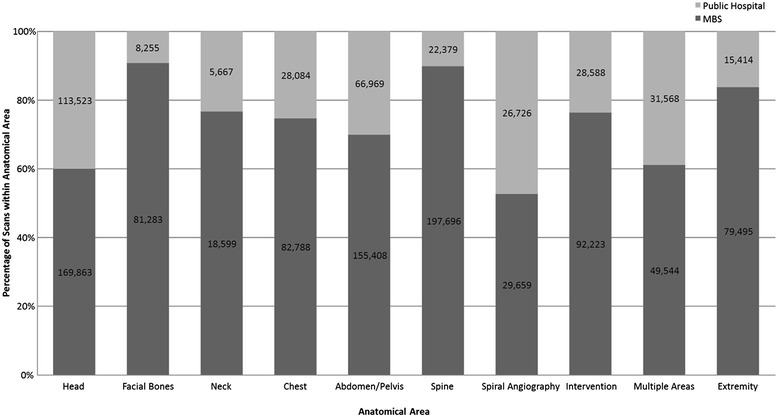
**Percentage of WA CT scans performed in each provider setting within each anatomical area from mid-2006 to mid-2012.**

Figure 1 presents the percentage of services performed in each provider setting for each anatomical area (including raw scan numbers) over the study period. The MBS setting provided at least 60% of all scans in each anatomical area, except for spiral angiography with 53% of scans in the MBS setting. The anatomical areas most dominated by the MBS were facial bones (91%) and spine (90%). Head CT was substantially the most commonly scanned anatomical area in the public hospital setting despite only accounting for 40% of all head CT scans in WA.

Table 2 shows age-standardised adult CT scan rates for each anatomical area by sex, provider setting and year. Three anatomical areas presented consistently higher CT scan rates in the MBS sector for both males and females: head, abdomen/pelvis and spine. In the public hospital setting, two areas showed substantially higher scanning rates: head and abdomen/pelvis; however, the head scan rate was distinctly higher than the abdomen/pelvis rate. Most anatomical areas saw small increases in scanning rates in both provider settings; however some areas declined slightly in the MBS setting (head, neck, spine). The largest increase from 2006/07 to 2011/12 occurred in MBS intervention CT scans, rising from 7.3 to 11.4 scans per 1,000 PY.

**Table 2:** **Age standardised Western Australian CT scan rates (scans per 1,000 persons) by provider setting, anatomical area and sex**

**Sex**	**Anatomical Area**	**2006/07**		**2007/08**		**2008/09**		**2009/10**		**2010/11**		**2011/12**	
		**MBS**	**Public Hospital**	**MBS**	**Public Hospital**	**MBS**	**Public Hospital**	**MBS**	**Public Hospital**	**MBS**	**Public Hospital**	**MBS**	**Public Hospital**
Male	Head	14.7	11.4	14.3	11.7	14.5	12.1	14.0	12.1	12.8	12.4	13.6	12.5
	Facial Bones	6.9	0.9	6.7	1.0	6.4	1.1	6.1	1.0	5.8	1.0	8.3	1.0
	Neck	1.5	0.7	1.7	0.6	1.8	0.7	1.7	0.6	1.6	0.7	1.8	0.7
	Chest	8.6	3.3	8.3	3.4	8.7	3.2	8.2	3.0	8.6	3.3	8.9	3.4
	Abdomen/Pelvis	13.4	6.5	13.9	7.0	14.4	7.3	13.8	7.1	14.0	7.7	15.0	8.5
	Spine	18.8	2.3	18.5	2.6	18.8	2.8	17.4	2.6	15.5	2.7	17.1	2.8
	Spiral Angiography	2.6	2.4	2.7	2.7	3.0	2.7	3.0	2.7	2.9	2.9	4.0	3.1
	Intervention	5.6	3.2	6.2	3.4	6.7	3.5	7.7	3.4	8.6	3.5	9.7	3.1
	Multiple Areas	3.8	3.4	4.2	3.2	4.8	3.4	5.1	3.4	5.3	3.5	6.0	3.6
	Extremity	7.8	1.7	7.5	1.9	8.2	2.0	7.9	1.8	7.3	1.7	7.9	1.8
Female	Head	21.0	9.6	20.8	9.6	20.4	10.6	19.4	10.3	17.8	10.9	18.6	11.2
	Facial Bones	9.7	0.6	9.5	0.5	8.9	0.7	8.6	0.6	8.2	0.6	11.1	0.7
	Neck	2.2	0.5	2.2	0.4	2.1	0.5	1.8	0.4	1.8	0.4	1.9	0.5
	Chest	8.0	2.2	7.8	2.3	8.1	2.4	7.6	2.1	7.6	2.2	7.8	2.4
	Abdomen/Pelvis	16.3	5.1	16.3	5.5	16.8	5.9	16.3	5.7	16.0	6.1	17.9	6.8
	Spine	22.9	1.5	22.7	1.7	23.0	1.9	21.1	1.8	18.7	1.9	20.6	1.8
	Spiral Angiography	2.2	2.2	2.5	2.5	2.7	2.3	2.8	2.4	2.8	2.7	3.8	2.9
	Intervention	8.9	2.1	9.2	2.4	9.6	2.3	10.9	2.4	12.2	2.3	13.1	2.2
	Multiple Areas	4.1	2.5	4.3	2.7	4.8	2.9	4.9	2.7	5.1	2.9	6.0	3.1
	Extremity	7.5	1.2	8.0	1.3	8.2	1.2	7.9	1.2	7.6	1.1	8.5	1.3
All	Head	17.9	10.5	17.6	10.7	17.4	11.3	16.7	11.2	15.3	11.6	16.1	11.9
	Facial Bones	8.3	0.7	8.1	0.8	7.6	0.9	7.4	0.8	7.0	0.8	9.7	0.8
	Neck	1.9	0.6	1.9	0.5	2.0	0.6	1.8	0.5	1.7	0.6	1.8	0.6
	Chest	8.3	2.8	8.1	2.9	8.4	2.8	7.9	2.6	8.1	2.8	8.4	2.9
	Abdomen/Pelvis	14.8	5.7	15.1	6.2	15.6	6.6	15.1	6.4	15.0	6.9	16.4	7.7
	Spine	20.8	1.9	20.6	2.1	20.9	2.4	19.2	2.2	17.1	2.3	18.8	2.3
	Spiral Angiography	2.4	2.3	2.6	2.6	2.9	2.5	2.9	2.6	2.9	2.8	3.9	3.0
	Intervention	7.3	2.6	7.7	2.9	8.2	2.9	9.3	2.9	10.4	2.9	11.4	2.7
	Multiple Areas	4.0	3.0	4.3	3.0	4.8	3.2	5.0	3.0	5.2	3.2	6.0	3.4
	Extremity	7.7	1.4	7.7	1.6	8.2	1.6	7.9	1.5	7.4	1.4	8.2	1.6

In the MBS setting, females predominantly showed scanning rates higher than males, except for chest, spiral angiography, extremities and multiple areas CT scans. In the public hospital setting males presented with higher rates than females without exception.

**Figure 2:**
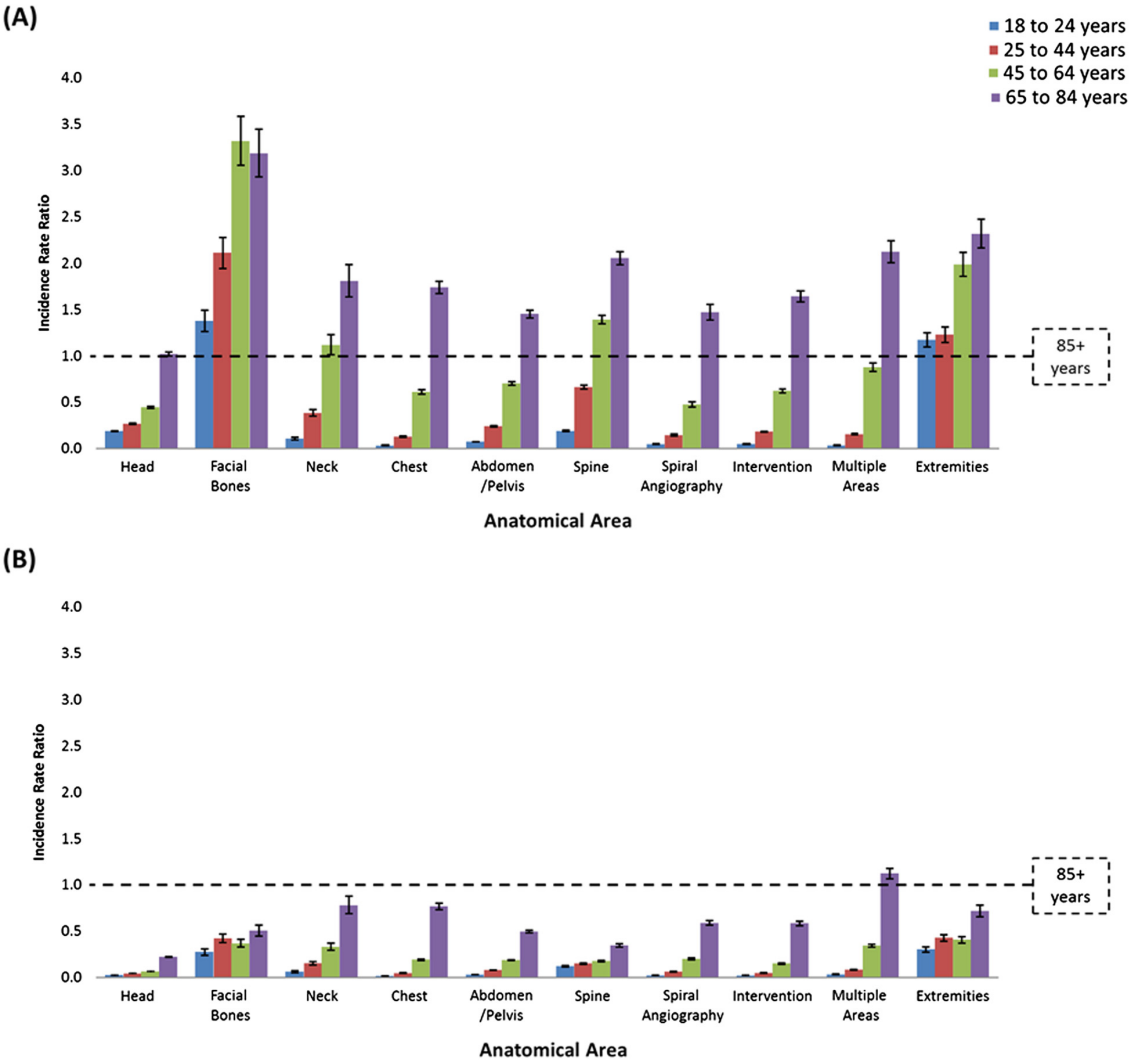
**Relative incidence rate ratios for (A) MBS and (B) Public Hospital CT scanning by anatomical area by age groups, adjusted for year and sex.**

Figure 2 presents the relative likelihood of a CT examination in each age group relative to patients aged 85+ years (IRR =1) by anatomical area (modelled seperately) in each provider setting. In the MBS setting, patients in age groups under 85 years were significantly more likely to undergo CT scanning of every anatomical area, except for head scans where there was no significant difference between the oldest two aged groups (Figure 2A). For the majority of anatomical areas in the MBS setting, patients aged 85+ years had the second highest scan likelihood; except for CT of the facial bones and extremities where patients 85+ years were least likely to be scanned. In the public hospital setting, patients aged 85+ years were significantly more likely to have a CT scan than any other age group in all anatomical areas, except ‘multiple areas’ where patients aged 64–84 years were more likely (Figure 2B).

## Discussion

In examining adult CT rates and the adjusted (i.e., independent) likelihood of receiving a CT scan in WA by provider setting, sex, age and anatomical areas across several years, our study provides a detailed picture of CT scan utilisation by adults in the Australian health care system. Importantly this study identifies differences in utilisation across the two provider settings previously assumed, but not quantified. Our study provides accurate estimates of adult CT utilisation rates in WA due to the coverage of both provider settings compared with previous estimates limited to only the MBS setting.

We observed an increase in CT scanning rates over six years across both provider settings in both sexes and in every age group except patients aged over 85 years. The most recent Australian CT scanning rates, published by the OECD is 2011 [2], were reported as 90.6 per 1,000 PY. In our study, the 2010/11 adult rate of CT utilisation was 125.4 per 1,000 PY. This is not surprising given that the OECD figures exclude the public hospital setting. However, a partial explanation could be differences in the range of ages covered by the rates, with this study restricted to the adult population aged 18 or more years.

Our study showed substantial differences in the number, trends and typology of CT scanning undertaken in the two sectors. A larger number and rate of CT scans was undertaken in the MBS setting compared with public hospitals. In each year of the study the crude female CT scan rate was higher than in males, but deeper analysis revealed the sexes primarily utilise CT in different settings (females in the private practices and males in the public hospital setting). Patients aged over 85 years in the public hospital setting were the most likely age group to have a CT in nine of the ten anatomical sites. Conversely, younger age groups were more prominently represented in the MBS setting, especially patients 65 to 84 years of age. The starkest contrast between the two sectors occurred in scans of facial bones and extremities, where patients aged over 85 years were the least likely group to be scanned in the MBS setting, but the most likely group in the public hospital environment. Overall, the MBS setting supported a more diverse range of CT services to a younger, more female patient base in greater volume than the public hospital setting.

Evaluation of CT utilisation is crucial for several reasons. Radiation from CT scans delivers a population burden, as well as benefit, and this radiation cost-benefit is not equal across different groups of patients. Unnecessary (i.e., non-evidenced based) use of CT also places an unjustified cost on the health system. Thus review of service utilisation patterns can inform policymakers of the impact payment structures or competing modality uptake has on population burden and benefit. There are policy responses available within each of these domains to ensure efficacy, minimising risks and optimising cost effectiveness.

### Radiation risks for patients

The linear-no-threshold model (i.e., no minimum safe level) is the prevailing theory of risk from radiation exposure [20]. As CT scanning develops new techniques, providing greater opportunity for utilisation, rigorous oversight and responsible access must reflect the principle of achieving radiation exposure that is as low as reasonably achievable (ALARA) [1,23,24]. The ALARA principle is primarily an application of common sense by medical personnel to: (a) minimise exposure time; (b) maximise distance from source; and (c) whenever possible, utilise shielding and barriers [1]. The ALARA principle is not only applicable at the patient level, but also at a systematic or population level.

Risks of radiation exposure to children are well publicised and inform a cautionary culture of radiation protection within paediatric services [8,9,23]. Radiation exposure risk is greater the younger an individual is at exposure [20]. However, while there is risk at exposure at all ages it does diminish, meaning when exposed to the same radiation middle-aged adults are at greater risk than older adults [20]. Additionally, females are at a greater risk from exposure than males at any age (not only due to cancers males are much less likely to develop, such as breast cancer) [20]. According the Committee on the Biological Effects of Ionizing Radiation's seventh report (BEIR-VII), the lifetime risk of all cancers for exposure to a single 0.1 Gray (Gy) radiation dose in females is 1.68 times higher than in males when exposed at age 20 years, 1.55 times higher if exposed at 30 years, 1.37 times higher at 40 years of age, 1.25 times higher at 50 years of age, 1.20 times higher at 60 and 70 years of age and 1.23 times higher at age 80 years [20]. Responsible rationing of high radiation imaging services delivers health system savings, not just in terms of utilisation rates and short-term expenditure; but also through a reduction in subsequent adverse outcomes such as radiation-induced cancer. Research from this team combining Australian MBS scanning records, collected CT dosimetry and the BEIR-VII exposure risk values identified a disproportionate burden risk of cancer incidence and mortality in females and younger adults [25].

Not only do the younger patients have greater time to develop cancer or are women more susceptible to ionising radiation burden, but particular anatomical sites carry differing radiosensitivities [17,26–28]. Application of the ALARA principle suggests the use of shields or barriers whenever possible. The prevalence of shield or barrier use in Australia by radiology services is unknown. Also of low prominence in discussions and research around medical radiation safety is the scan length of CT scans. While anatomical start and stop points will be used to guide scanning practices, machine over-scan and operator decisions can result in the inclusion of radiosensitive organs not intended for study or examination, such as the thyroid or liver during a chest scan that extends beyond the apices of the lungs or diaphragm. Inclusion of start and stop points for scans and the use of shields or barriers within industry guidelines or regulations could reduce unnecessary doses from certain scan types.

### Guidelines for medical radiation exposure

In keeping with the ALARA principle, DRLs have been introduced in various countries to afford CT providers an indication of acceptable dosimetry values. Australia’s own DRLs for adult CT, set by the Australian Radiation Protection and Nuclear Safety Agency (ARPANSA) [29], are relatively new compared with the UK and European Union guidelines [28,30]. Participation of practices in submitting data to DRL surveys and audits of practice performance against the DRLs are undertaken at the discretion of each provider in most nations including Australia [28–30]. Previous research in WA revealed substantial variation in CT scanning protocol settings and subsequent estimated radiation doses across provider settings [7]. Using a DRL survey methodology across a small sample of providers in WA for a selection of common CT scanning protocols, it was found these WA providers used higher dose scans than reported internationally for every scanning scenario [7].

With expanded technological capacities of CT machines and database systems, such as Picture Archiving and Communication Systems and Digital Imaging and Communications in Medicine fields, automatic collection of CT machine settings and dose outputs is now feasible. Accessing dose information from these sources is more rigorous and less prone to selection biases or judgements around ‘standard’ scanning practices the current DRL survey methodologies risk [31]. The barriers to participation in ongoing DRL surveys, protocol and dose audits are primarily motivational or cultural rather than technical. Modern CT and hospital information systems are capable of generating comprehensive databases on the scanning habits of providers and practice networks, rather than methodologically limited surveys prone to selective sampling, participation biases and low industry participation rates [32].

### Unnecessary use of CT

Incentives within the Australian health system are different depending upon the setting. The MBS operates as a fee-for-service system, where MBS items include a description that determines the capacity for a given service’s eligibility for payment to the provider of a set fee (schedule fee) from the federal government. Essentially, if a service does not fit the description of an MBS item there will be no subsidisation available from the federal government. Providers are able to charge the patient service fees in excess of the schedule fee; ultimately the difference is borne by the patient. Funding of public hospital services is via a separate mechanism negotiated as complex state and federal government health care funding agreements and is not based upon a fee-for-service model; leaving public hospital patients discharged without fee. Australian public hospitals are subject to more diffuse incentives regarding service delivery. The primary focus of recent health care funding agreements has been placing downward pressure on growing expenditure trends.

In order to restrict utilisation to indications shown to be cost-effective, the MBS has recently begun to incorporate specific clinical indications in the MBS item descriptors. This has been largely confined to newer technologies, such as Positron Emission Tomography (PET) and Magnetic Resonance Imaging (MRI). Narrowing the scope for use of CT is one possible mechanism to provide greater assurance of appropriate use. However, to re-write MBS item descriptions and establish clinical parameters for a scan to be performed, where none was included previously, requires substantial engagement with medical and imaging stakeholders.

Similar to other health systems, Australian GPs are able to refer patients for CT scans (performed overwhelmingly in MBS subsidised private services). There are concerns not all GPs appreciate the levels of radiation dose involved in a CT scan. Several studies have found specialists and GPs inaccurately estimate specific or relative CT doses in comparison to chest x-rays [33–37]. A systematic review of physician surveys found CT dose underestimations in a very high proportion of physicians (60-99%) [33]. The same systematic review noted attendance of a radiation protection course was positively associated with better knowledge of CT doses in two of five surveys that asked subjects about their education or experience [33].

Inaccurate perceptions among the group of doctors most accessible to the public and capable of referring patients to CT scans subsidised with unrestrictive MBS items runs the risk of inappropriate use. Several studies have found as much as 30% of all imaging tests are inappropriate in some fashion [38–40]. In some instances no imaging test is warranted at all, but more often are the situations where an alternative modality could have been utilised with lower or no radiation burden such as ultrasound, a plain radiograph or, MRI imaging. While the different modalities are not completely interchangeable, there is evidence use of these alternative services can offset some of the workload borne by CT machines and, by so doing, reduce the population radiation exposure burden and consequent cancer incidence [41]. Furthermore, previous publications have argued the economic cost of the radiation dose saved by using MRI rather than CT should be factored into cost-benefit assessments of MRI machines [42,43].

Improved training of GPs could potentially reduce inappropriate CT scans and use of streamlined referral schema (‘diagnostic imaging pathways’) can assist the implementation of the ALARA principle [19]. More extreme measures such as removing GP referral for CT scanning would not only meet considerable political resistance, but would also be inconvenient to patients and increase demand for medical specialist services.

### Strengths and limitations

The data collections used in this study were robust routinely collected administrative records of CT services used in both the public hospital and MBS settings. A significant strength of this study is the use of both public hospital and MBS data to capture the whole health care system rather than the limited examination of only MBS CT data in international comparisons, such as the OECD and UNSCEAR reports [17,18]. The ABS census data provide the best available population figures for the estimation of rates and relative risk. WA also provides a representative sample of the national population [44].

A limitation to the study was the lack of clinical information within the data sources beyond general demographic information and the standard descriptions associated with CT scan codes. This necessarily limits our capacity to comment on the trends observed, especially regarding systematic differences in clinical indication and patient medical history, to broader more systematic issues regarding economic incentives and radiation risk management regulation. Our study was also limited to adults and in doing so the MBS aggregate data for 15 to 24 year olds was cut by proportion to an 18 to 24 year old group; this assumes a consistent level of CT use for each year group in this age group. Paediatric radiology operates within a different clinical culture, especially within the hospital system, limiting the applicability of the results of this paper to children’s CT services [3]. We are unable to provide an accurate comparison of WA with other states in terms of hospital CT services due to the diversity of state health system organisations. Each Australian state is also responsible for CT machine licencing with limited uniformity of reporting of machine numbers, making a comparison of CT machine prevalence between states or nationally impracticable.

## Conclusion

Western Australian private practice CT scans constituted the bulk of CT services from mid-2006 to mid-2012. The public hospital sector CT scans were more likely to be performed on males; patients aged over 85 years and were predominantly head scans, whereas private practices tended to scan females and relatively younger patients with a diverse range of CT scan types, including those capturing anatomical sites with greater radiosensitivity. These two sectors present substantially different risk profiles with respect to CT utilisation. Private radiology providers, operating in the context of potentially contradictory commercial and radiation protection signals, perform the majority of CT scans with substantial subsidisation from the federal government. Appropriate levels of regulation, review and rationing of access are essential for any medical technology, especially with the potential for public health burden present in CT scanning. Australia has several avenues for expansion or refinement of acceptable dose levels, CT services eligible for funding, alternative diagnostic modalities and physician radiation dose training to ensure individual and population medical radiation dose is as low as reasonably achievable.
